# Eudragit^®^ FS Microparticles Containing Bacteriophages, Prepared by Spray-Drying for Oral Administration

**DOI:** 10.3390/pharmaceutics15061602

**Published:** 2023-05-27

**Authors:** Emilie Tabare, Tiffany Dauchot, Christel Cochez, Tea Glonti, Céline Antoine, Fanny Laforêt, Jean-Paul Pirnay, Véronique Delcenserie, Damien Thiry, Jonathan Goole

**Affiliations:** 1 Laboratory of Pharmaceutics and Biopharmaceutics, Faculty of Pharmacy, Université Libre de Bruxelles, 1050 Brussel, Belgium; tiffany.dauchot@ulb.be (T.D.); jonathan.goole@ulb.be (J.G.); 2 Laboratory for Molecular and Cellular Technology, Queen Astrid Military Hospital, 1120 Brussels, Belgium; christel.cochez@mil.be (C.C.); tea.glonti@mil.be (T.G.); jean-paul.pirnay@mil.be (J.-P.P.); 3 Food Science Department, FARAH and Faculty of Veterinary Medicine, University of Liège, 4000 Liège, Belgium; celine.antoine@uliege.be (C.A.); fanny.laforet@uliege.be (F.L.); veronique.delcenserie@uliege.be (V.D.); 4 Bacteriology, Department of Infectious and Parasitic Diseases, FARAH and Faculty of Veterinary Medicine, University of Liège, 4000 Liège, Belgium; damien.thiry@uliege.be

**Keywords:** bacteriophage, colon-targeting, spray-drying, Eudragit^®^ FS, tablets, capsules, phage-therapy

## Abstract

Phage therapy is recognized to be a promising alternative to fight antibiotic-resistant infections. In the quest for oral dosage forms containing bacteriophages, the utilization of colonic-release Eudragit^®^ derivatives has shown potential in shielding bacteriophages from the challenges encountered within the gastrointestinal tract, such as fluctuating pH levels and the presence of digestive enzymes. Consequently, this study aimed to develop targeted oral delivery systems for bacteriophages, specifically focusing on colon delivery and employing Eudragit^®^ FS30D as the excipient. The bacteriophage model used was LUZ19. An optimized formulation was established to not only preserve the activity of LUZ19 during the manufacturing process but also ensure its protection from highly acidic conditions. Flowability assessments were conducted for both capsule filling and tableting processes. Furthermore, the viability of the bacteriophages remained unaffected by the tableting process. Additionally, the release of LUZ19 from the developed system was evaluated using the Simulator of the Human Intestinal Microbial Ecosystem (SHIME^®^) model. Finally, stability studies demonstrated that the powder remained stable for at least 6 months when stored at +5 °C.

## 1. Introduction

Antibiotic resistance is increasing worldwide [1]. The use of lytic bacteriophages is one of the promising alternative or additional tools to treat bacterial infections [2,3]. In addition, bacteriophages may also be used in prophylaxis, which is particularly useful for patients with chronic diseases, immunocompromised patients, or those that will be subjected to surgery or hemodialysis to avoid infections by antibiotic-resistant pathogens and complications for these weakened patients [4]. Among the various administration routes, oral delivery is usually the most convenient and most likely to be accepted by patients [5]. Oral dosage forms may be used to treat local pathologies of the gastrointestinal tract (GIT) [6], as well as urinary tract infections (UTIs), including multidrug-resistant infections [7]. In uncomplicated UTIs, *Pseudomonas aeruginosa* is not frequently detected (7–15%). In contrast, it is more frequently observed in complicated UTIs [8], showing a higher prevalence of antimicrobial resistance and a greater propensity to form biofilms on medical devices than *E. coli* or *K. pneumoniae* [9]. Moreover, the non-invasive administration of an oral dosage form is very suitable for immunocompromised patients, who are more susceptible to infection by opportunistic bacterial pathogens, such as *P. aeruginosa*, and are at risk of life-threatening infections (e.g., Typhlitis or rectal abscesses in neutropenic patients, necrotizing enterocolitis in premature infants, and Shanghai fever, …) [10,11]. Moreover, the decolonization of *P. aeruginosa* from the intestine to prevent invasion from the gut is a crucial objective [12]. Indeed, studies have shown that the gastrointestinal tract is the biggest *P. aeruginosa* reservoir of the body [13] and ICU patients who exhibit *P. aeruginosa* colonization suffer from a significantly higher mortality rate than those who do not [14]. Reports indicate that decontamination of the gastrointestinal tract can lead to reduced rates of ICU-acquired infection [15,16,17], reinforcing the fact that *P. aeruginosa* colonization of the gut is a precursor to invasive infection. A prospective randomized trial has confirmed the significance of intestinal *P. aeruginosa* in the mortality of critically ill patients [18]. Unfortunately, bacteriophages are often sensitive to extreme pH values, such as those found in the stomach. Therefore, acidity neutralizers are often advised and used to improve the stability of the bacteriophages when orally administered (e.g., Ranitidine and Omeprazole) [19].

Different types of excipients, as well as several strategies of formulation, to protect bacteriophages from acidic and enzymatic degradations in the stomach and at the beginning of the small intestine were already described in the scientific literature [20]. The most commonly used excipient for the encapsulation of bacteriophages is alginate, which may be added after extrusion coupled with gelation [21,22,23,24,25]. The use of alginate alone was described to be insufficient to protect bacteriophages from stomach conditions. However, the addition of calcium carbonate as an antacid excipient to the alginate microspheres significantly improved the survival of encapsulated bacteriophages [22]. Excipients, such as mannitol [23], pectin [26], and chitosan [25], are also described to increase the gastroprotection of alginate. Whey proteins (e.g., milk proteins) were also used in addition to alginate and it was shown that the resulting microspheres provided better preservation of bacteriophage activity than the chitosan-based alginate formulations [22,27,28]. However, the use of milk proteins of animal origin could induce allergy or intolerance in the patients. Moreover, some bacteriophage–bacteria interactions have been reported to be inhibited by bovine whey proteins [29]. On the other hand, dried preparations of microencapsulated bacteriophages are desirable for prolonged storage, convenience of transportation, and delivery. Trehalose, sucrose, maltodextrin, skim milk [22], and mannitol [23] were used as desiccant protectants.

The efficacy of poly(DL-lactide-glycolide) and poly(vinyl alcohol) derivatives as delivery systems was evaluated by Puapermpoonsiri et al. in a water-in-oil-in-water double emulsion coupled with freeze-drying [30]. Despite the biodegradability of their formulation, the process was too expensive, the formulation had a poor shelf-life (e.g., after a period of 7 days, no further lytic activity was observed at either 4 or 22 °C), and the loss of lytic activity was quite high [30]. González-Menéndez et al. encapsulated bacteriophages in niosomes and transferosomes [31]. Another recently described method to encapsulate bacteriophages is based on microfluidic technology [32,33,34]. However, such methods are quite expensive and difficult to scale up [35]. There is a need for scalable low-cost methods, such as spray-drying, to produce stable oral dosage forms to deliver bacteriophages in the GIT. Spray-drying was previously used to produce stable bacteriophage-containing powders in sugar-based formulations [36,37,38,39,40]. In contrast, the number of studies describing spray-drying to produce bacteriophage-containing powders with pH-responsive characteristics mediated by Eudragit^®^ derivatives is relatively low. Indeed, to the best of our knowledge, only a couple of such studies, using Eudragit S100^®^, can be found in the literature [35,41]. Stanford et al. encapsulated four bacteriophages (wV8, rV5, wV7, and wV11) active against *Escherichia coli* 0157: H7 using a rotary atomizer. The encapsulation of the bacteriophages resulted in a loss of activity of about 1 log_10_ after the process and after exposure to a pH of 3.0 for 20 min, resulting in an average recovery of 13.6% of the bacteriophages after their release at pH 7.2. In a second study, Vinner and co-workers encapsulated the Felix O1 bacteriophage (*Ounavirinae*) against *Salmonella enterica* using a pneumatic atomizer. Five different formulations were developed to protect the bacteriophage against stomachal acidity. The formulations were as follows: 4% trehalose (1); 3% *w*/*v* of Eudragit S100^®^ (2); 3% Eudragit S100^®^ and 2% trehalose (3); 2% Eudragit S100^®^ and 1% trehalose (4); 2% Eudragit S100^®^; and 4% *w*/*v* trehalose (5). For formulation (1), containing trehalose only, the authors indicated that there was no measurable loss of bacteriophage activity upon spray-drying. For formulation (2), containing Eudragit^®^ S100 only, a loss of 4 log_10_ after spray-drying was observed. In general, formulations without trehalose were not able to protect the bacteriophage from desiccation during the spray-drying process. For formulations containing both trehalose and Eudragit^®^ S100, the authors indicated that there was no difference with the formulation containing trehalose only. After spray-drying, the Felix O1 bacteriophage was encapsulated in microparticles based on different ratios of Eudragit^®^ S100 and trehalose. These microparticles were submitted for 2 h to a simulated gastric fluid at pH 2. The reduction of the bacteriophage activity reached at least 2 logs_10_ after 2 h in an acidic medium, depending on their formulations.

To the best of our knowledge, the spray-drying of bacteriophage LUZ19 using Eudragit^®^ FS30D to protect it from stomachal acidity has not been reported before in the scientific literature. The colonic-release polymer was selected to avoid a potential early release of the phage as it is characterized by the highest pH of dissolution among the Eudragit^®^ derivatives. Indeed, the stomach and some regions of the small intestine are characterized by a pH lower than 7.0, which could affect the in vivo activity of the phage. 

Moreover, to develop an innovative formulation, Eudragit^®^ FS30D, poly(methyl acrylate-co-methyl methacrylate-co-methacrylic acid), was selected as the type S was already described in the literature. In addition, the SHIME^®^ model was used to simulate the release of the bacteriophages in the GIT. Finally, a deep stability study of the bacteriophage activity, as well as its protection from an acidic environment, was also performed at 5 ± 2 °C or 25 ± 2 °C and 60% relative humidity (RH); 30 ± 2 °C and 65% RH; and 40 ± 2 °C and 75% RH.

In this present study, our primary objective was to develop colon-targeted oral delivery systems, based on the Eudragit^®^ FS derivative, which provide a loss of bacteriophage activity lower than 2 logs_10_. Moreover, the powder must be able to be conserved at fridge temperatures (2–8 °C) without losing its acid protection property or bacteriophage activity. Finally, the powder could be filled in a capsule or mixed with conventional excipients to produce tablets.

## 2. Materials and Methods

### 2.1. Materials

The bacteriophage named LUZ19, which is a *P. aeruginosa* bacteriophage classified as a podovirus, with propagation strain PAO1K was used as a model bacteriophage (2.0 × 10^11^ pfu/mL). Professor Rob Lavigne of KU Leuven, Belgium provided the LUZ19 bacteriophage while the Queen Astrid Military Hospital in Brussels, Belgium supplied the propagation strain PAO1K. A prior study provided a comprehensive description of bacteriophage LUZ19 [40].

D-(+)-trehalose dihydrate and L-isoleucine of non-animal origin were purchased from Sigma-Aldrich (Overijse, Brussels). Eudragit^®^ FS 30D, an aqueous dispersion of anionic copolymer based on methyl ACRYLate, methyl methACRYLate, methACRYLic acid, as well as PlasACRYL^®^ T20, and Aerosil^®^ 200 VV Pharma, were purchased from Evonik (Darmstadt, Germany). PlasACRYL^®^ T20 is a 20% emulsion made of sliding agent and plasticizer. It is composed of water, glycerol monostearate, triethyl citrate, and polysorbate 80 and is designed to be used exclusively with the Eudragit^®^ FS30D. 

Spray-dried mannitol (Pearlitol^®^ 100), used as diluent, microcrystalline cellulose (MCC 102), binder and croscarmellose sodium (Solutab A), and superdisintegrant, were generously offered by Roquette (Lestrem, France). Medium viscosity hydroxypropyl cellulose (HPC, Nisso HPC-M, Nippon Soda, Tokyo, Japan) was also used. Ultrapure (ELGA) water (>18.2 MΩ) was used as dispersant.

The capsules used were Capsugel^®^ Vcaps^®^ Plus, sizes 00, 0, and 1 (Lonza, Verviers, Belgium). 

### 2.2. Methods

#### 2.2.1. Bacteriophage Stock Preparation

LUZ19 bacteriophage stocks were prepared using the double agar overlay method as described in a previous paper with the *P. aeruginosa* strain PAO1 K [40,42].

#### 2.2.2. Temperature and pH Stability of Bacteriophage Activity

Temperature stability was assessed by incubating 1 mL of 10^8^ PFU/mL bacteriophage samples at different temperatures (25 °C, 37 °C, 45 °C, and 60 °C). For pH stability, 100 μL of 10^9^ PFU/mL bacteriophage samples were diluted in 900 μL of PBS at different pH levels (2, 4, 6, 8, 10, and 12). All samples were incubated for 1 h and subsequently titrated in biological triplicate [43]. 

#### 2.2.3. Spray-Drying

The bi-fluid nozzle was used with formulations containing different D-(+)-trehalose dihydrate with L-isoleucine (63.3% *w*/*w* and 36.7% *w*/*w*, respectively) and Eudragit^®^ FS-30D with PlasACRYL^®^ T20 (90.9% *w*/*w* and 9.1% *w*/*w*, respectively) weight ratios (Table 1). The percentages of D-(+)-trehalose dihydrate with L-isoleucine were set according to the optimal formulation of a previous study with LUZ19 [40] and the percentages of Eudragit^®^ FS-30D and PlasACRYL^®^ T20 were set according to the recommendations of Evonik. Using a Büchi Mini Spray-dryer B-290 (Flawil, Switzerland) and a Büchi Dehumidifier B296 (Flawil, Switzerland), the formulations (Table 1) were spray-dried as 5% *w*/*v* aqueous solutions with 1% *v*/*v* of bacteriophages. The formulation was fed continuously at a rate of 2 mL/min, with an aspiration rate of 35 m^3^/h, Tin of 80 °C, and spray gas flow of 819 L/h. After processing, each powder was stored in type 1 clear pharmaceutical-grade glass amber vials, sealed with ultra-pure chlorobutyl-isoprene rubber stoppers and aluminum caps, at typical ICH storage conditions. 

#### 2.2.4. Bacteriophage Stability Testing

For each formulation, the stability of bacteriophages was assessed under acidic (pH 2) and neutral (pH 7.4) conditions (*n* = 3). Ten mL of HCl 0.037% were added to vials containing 150 mg of powder. The particles were in contact with the acidic media for 2 h before being filtered under vacuum with Durapore^®^ 0.22 μm polyvinylidene difluoride (PVDF) membrane. Then, they were placed in vials to be dispersed in 10 mL of DPBS (pH = 7.4) w/o Ca^2+^ and Mg^2+^. Samples (*n* = 3) were also immediately reconstituted in 10 mL of DPBS as the positive control. 

The spot testing on agar overlay technique was used to determine the number of viable bacteriophages (PFU/mL) in the powder samples as described in a previous study [40,44].

For each step, titration triplicates were conducted to ensure uniformity of the batch, and biological triplicates were conducted for the analytical method.

#### 2.2.5. Production Yield

The production yields were determined by weighing the collected powder from the spray dryer collection vessel after spray-drying and then calculating it as a percentage of the weight of the dry materials that were initially added to the feed solution (% *w*/*w*).

#### 2.2.6. Scanning Electron Microscopy

To examine the shape of the desiccated particles, a Hitachi SU8020 SEM-FEG microscope (located in Chiyoda, Tokyo, Japan) was used. The samples were first affixed to a round platform using an adhesive carbon tab and subsequently coated with 2.5 nm of platinum/palladium (Pt/Pd) using a Leica ACE600 metallizer before being subjected to imaging. The images were captured at 5 kV using the secondary electron detector SE (L), which is located within the chamber. The SE (U) detector located in the column was used to obtain images at higher magnifications, using a voltage of 2 kV. The magnifications used in acquiring the images were 1000, 5000, 30,000, and 50,000×.

#### 2.2.7. Thermal Analysis

The TA instruments, Q500 apparatus and Universal Analysis 2000 (version 4.4A) software, both manufactured by TA Instruments in Belgium, were used to evaluate the quantity of residual water through thermogravimetric analysis (TGA). The process involved placing around 10 milligrams of powder on a platinum cup and heating it at a rate of 10 °C/min over a temperature range of 30 to 200 °C. The weight loss between the powders at 30 °C and 120 °C was used to define the residual moisture content (RMC).

The evaluation of the glass transition temperature (Tg) was performed through the use of a DSC Q2000 instrument and Universal Analysis 2000 (version 4.4A) software (TA Instruments, Asse, Belgium). Differential scanning calorimetry (DSC) analysis was conducted while purging with nitrogen. To start the analysis, powder samples ranging from 2 to 4 milligrams were placed inside a Tzero hermetic aluminum pan, which was then sealed. Subsequently, the samples underwent a heating process with a heating rate of 10 degrees Celsius per minute, covering a temperature range of −50 to 150 °C.

#### 2.2.8. Powder X-ray Diffraction

Powder X-ray diffraction (PXRD) utilizing an X-ray diffractometer (D8 Advance Eco Bruker^®^, Madison, WI, USA), which possessed a one-dimensional silicon detector (LynxEye, Bruker AXS, Billerica, MA, USA) and employed Cu-Kα radiation (1.54 Å; 40 kV × 25 mA) was used. The data obtained were collected within the angular range of 4–40° 2θ. The step size was set at 0.02° and the dwell time at 2 s. The percentage of the amorphous form was derived by calculating 100% minus the content of the crystalline phase present in the powder, which was determined using the surface area ratio method.

#### 2.2.9. Particle Size Distribution

To determine the particle size distribution of the particles, a Malvern Mastersizer^®^ 3000 with an Aero S dry powder disperser unit (Malvern Instruments Ltd., Worcestershire, UK) were used. Samples (triplicates) were placed into the hopper of the disperser. A vibration level of 75% and a pressure of 4 bar were applied during the measurement.

#### 2.2.10. Determination of Density, Porosity, and Flowability of the Powder

Particle density (ρ*P*) was measured using a helium gas pycnometer Ultrapyc 5000 equipment (Anton Paar, Graz, Austria), according to the procedure described in the European Pharmacopeia [Ph.Eur.10.0,20242(01/2010)]. Approximately 10 g of the samples were weighed (in triplicate) and transferred to the 10 cm^3^ sample holder. A pressure of 10 psi was used at a temperature of 20 °C, with true density calculated in g/cm^3^. The results were obtained from fifteen measurements of volume and density. Loose bulk density (ρ*L*) was measured by using 10 mL plastic graduated cylinder. After passing through a 0.5 mm sieve, the powder is poured into a 10 mL stemmed glass, avoiding any shaking. According to the European Pharmacopeia [Ph.Eur.10.0,20934(04/2019)], the loose bulk density considers the density of the powder particles but also the interparticle void volume. It is calculated as the ratio between the mass of powder in the stemmed glass and the volume it occupies there and is expressed in g/mL, g/cm^3^, or kg/m^3^. Tapped bulk density (ρ*T*) was measured after the 1250 mechanical tapping of a graduated cylinder of 10 mL containing the powder sample. The device used was a Stampvolumeter STAV2003, with a tap height of 3 mm. The value of 1250 taps was chosen because it corresponded to the number of taps necessary to reach a constant volume of powder [45]. Porosity (ε) was calculated by using the relationship between tapped bulk ρ*T* and ρ*P*. Carr index (CI) was used for representing flowability of powders as described in the European Pharmacopeia [Ph.Eur.10.0,20936 (01/2010)]. CI was calculated using the tapped bulk density (ρ*T*) and the loose bulk density (ρ*L*). CI was classified as the following: <10 very good, 11–15 good, 16–20 fair, 21–25 passable, 26–31 bad, and >32 very bad [45]. Hausner ratio (HR) was used for representing cohesiveness of powders. HR was calculated as the ratio of ρ*T* and ρ*L*. HR was classified as the following: <1.19 low, 1.19–1.34 intermediate, and >1.34 high [45]. To be considered as having good flowability, the CI must be lower than 15% and the HR must be lower than 1.18.

#### 2.2.11. Mixing for Tableting

The mixing of the powders was performed gradually, as described below (Table 2). The spray-dried powder comprising the bacteriophages was first mixed with colloidal silicon dioxide after which the so-called “sandwich” mode of incorporation was applied. This was performed in a glass container where the total volume of the powder represents 40–60% of the container volume, with half of the microcrystalline cellulose and mannitol first deposited, then the premix with croscarmellose and hydroxypropyl cellulose, and then the other half of microcrystalline cellulose and mannitol. The powder was mixed for 15 min with a Turbula^®^ Mixer (WAB, Muttenz, Switzerland) at 33 rpm. Magnesium stearate was added at the end of the mixing process for 1 min. 

#### 2.2.12. Uniformity of Capsules

The capsules were manually filled by the hand-operated method commonly used in compounding pharmacies.

The uniformity of the capsules was assessed as described in the European Pharmacopoeia [Ph.Eur.10.0,20940(04/2017)]. Twenty capsules were taken at random and weighed.

#### 2.2.13. Tableting

Tablets were prepared by direct compression. The mixtures were automatically fed into the die of an instrumented single-punch tableting machine (Korch, Berlin, Germany) to produce tablets containing encapsulated bacteriophages using 7 mm concave-faced punches and dies.

#### 2.2.14. Tablet Tests

Mass uniformity of tablets was tested according to the European Pharmacopeia [Ph.Eur.10.0,20940(04/2017)]. Twenty units taken at random were weighed and the mean mass and standard deviation were determined. The resistance to crushing of tablets was evaluated according to the European Pharmacopeia [Ph.Eur.10.0,20908(01/2008)] with a hardness tester (Computest, Kreamer Gmbh, EL Ektronik, Darmstadt, Germany) on 10 tablets. Friability of tablets (Ph.Eur.10.0,20907501/2010) was carried out on 20 tablets by performing 100 rotations of the drum at 20 rpm. The friability was expressed as the percentage of mass loss. The authorized limit loss was 1% *w*/*w*. Disintegration tests were performed according to Ph.Eur.10.0,20901(01/2022). The used apparatus consisted of a basket containing 6 tubes (1 tablet per tube), open at both ends, and containing a wire mesh (2 mm mesh) at the lower part. The basket was immersed in a one-liter cylindrical container containing 0.1 N HCl. The temperature was set at 37 ± 1 °C.

#### 2.2.15. Simulator of the Human Intestinal Microbial Ecosystem (SHIME^®^)

To determine the release profile of formulated LUZ19 bacteriophages in the colon, a modified Simulator of Human Intestinal Microbial Ecosystem (SHIME^®^), i.e., an in vitro model of the gastrointestinal tract, was used. For this purpose, the release profile of formulation F4 was assessed, in triplicate, with three bioreactors. These fermenters were placed on magnetic stirrers (to mimic the dynamic of the gastrointestinal tract) and maintained at a temperature of 37 °C. They were filled with 140 mL of a broth called feed containing pectin, xylan, glucose, potato flour, arabinogalactan, extract of yeast, peptone, mucin, and cysteine provided by ProDigest (Ghent, Belgium), adjusted at pH 2 (with HCl 37%) and were connected to a synthetic pancreatic juice, PJ, containing sodium hydrogen carbonate, pancreatin, and bile salts provided by ProDigest (Ghent, Belgium). The pH of the reactors was adjusted using pH probes that automatically triggered the distribution of acidic (HCl 0.5 M) or basic (NaOH 0.5 M) solutions (provided by ProDigest (Ghent, Belgium)) to maintain a specific pH range and allowed for the monitoring of the pH in real- time.

Briefly, the formulated bacteriophage (1020 mg at ~2.0 × 10^6^ PFU/mg) was discharged in the different fermenters filled with feed (three per tested formulation). After incubation of the bacteriophage in the feed for 2 h, 60 mL of PJ was automatically added in each reactor for 15 min (which gradually conducted the solution to pH 7). After the entire PJ distribution, the pH of the bioreactors was forced at pH 7.4. Samples were taken after reaching pH 5, 6, 7, and 7.4. Once pH 7.4 was reached, three other samples were taken after 45 min, 120 min, 240 min, and 300 min. All samples were filtrated using 0.2 µm syringe filters (ref.514-0073, VWR, Leicestershire, UK) and then tenfold serially diluted in PBS (in triplicate) for the bacteriophage’s titration. Drops of 2 μL on the different dilutions were plated on enriched LB agar Petri dishes (with 1 mM MgSO_4_ and 1 mM CaCl_2_) on a soft-agar *Pseudomonas* overlay and heated at 37 °C for 12 h. After incubation, the plaques of lysis were counted to calculate the concentration of the delivered bacteriophages in the SHIME^®^ model.

#### 2.2.16. Stability Study

Stability testing was performed according to the ICH harmonized guidelines, as described in the Q1A(R2) document [46]. Stability testing was conducted on the bacteriophage formulations packaged in sealed vials for 3 months and tests were performed after 1 day, after 1 and 3 months, at 4 ± 2 °C; 25 ± 2 °C and 60% RH; 30 ± 2 °C and 65% RH; and 40 ± 2 °C and 75% RH in climatic chambers (Weiss, Loughborough, UK).

#### 2.2.17. Statistical Analysis

Statistical analysis was carried out using Graphpad™ version 9 (San Diego, CA, USA). Two-sample *t*-tests were performed (*n* = 3), with reporting of *p* ≤ 0.05 as statistically significant. Where multiple tests were conducted, the value of alpha was adjusted using the Bonferroni correction. Error bars represented a single standard deviation for the mean values of the replicates.

## 3. Results and Discussion

The spray-drying process is based on the use of relatively high temperatures. It is known that the thermal inactivation of bacteriophages is usually negligible if the outlet temperature is maintained under 40 °C [20]. Moreover, their inactivation due to their desiccation may be avoided using specific excipients, such as sugars or polyol derivatives, which may replace hydrogen-bonded water to preserve the activity of the bacteriophages. For instance, due to its high Tg (115 °C in its anhydrous form), as well as due to its non-reducing properties, D-(+)-trehalose is widely used in the spray-drying of bacteriophages [36,37,38,40,47]. Indeed, the reducing functional groups of other sugars, such as lactose, may damage the integrity of the bacteriophages, leading to their inactivation. In addition to the use of sugars, amino acids are commonly used to improve the dispersibility of dry microparticles or to protect them from the deleterious effects of residual moisture by creating an outer shell around the dried particles. It was previously demonstrated that L-leucine was particularly efficient [48]. However, Mah et al. recently conducted a study to compare the effects of L-leucine and L-isoleucine in reducing moisture-induced changes in spray-dried D-(+)-trehalose formulations. They demonstrated the greater ability of L-isoleucine to overcome elevated humidity compared to L-leucine for samples of the same concentration. Therefore, in this work, D-(+)-trehalose and L-isoleucine were selected to preserve the activity of LUZ19 and improve the dispersibility of the particles, respectively [49].

On the other hand, enteric coatings are used in oral drug delivery systems to protect a drug from the acidic environment of the stomach by preventing its release until it reaches the small intestine [50]. Indeed, without the presence of a colonic polymer (e.g., Eudragit^®^ S100), it was shown that the formulation did not protect bacteriophages from acidic degradation [51]. Polymeric materials, such as Eudragit^®^ derivatives, cellulose acetate phthalate, and hydroxyl propyl methyl cellulose phthalate/acetate succinate, are commonly utilized due to being unionized and insoluble at a low pH, and ionized and soluble in the higher pH of the GIT [52]. Nevertheless, Eudragit^®^ derivatives appear to be the most commonly used family of polymers to be used in spray-drying to develop targeted-release drug delivery systems intended for oral administration [50]. However, there are very few studies describing the atomization of bacteriophage-loaded formulations using Eudragit^®^ derivatives. Indeed, to the best of our knowledge, there are only a couple of studies describing the use of Eudragit^®^ S100, a functional delayed-release polymer for colon delivery and gastrointestinal targeting that dissolves at pH 7.0 [35,41]. Several polymers may be considered for colonic delivery. A gamma scintigraphy study in humans demonstrated the higher effectiveness of Eudragit^®^ FS, compared to Eudragit^®^ S, to promote the release of diclofenac sodium in the colon [53]. This result was taken into consideration by selecting an adequate colonic-targeting polymer in this study to avoid the inactivation of bacteriophages, as well as to deliver a stable bacteriophage-loaded formulation to the infection site. In addition to Eudragit^®^ FS, PlasACRYL^®^T20 was also added in the polymeric dispersion to reduce the phenomena of agglomeration and clogging of the nozzle during long runs of spray-drying, as occurring in continuous processes.

### 3.1. Influence of Temperature and pH on Bacteriophage Activity

**Figure 1:**
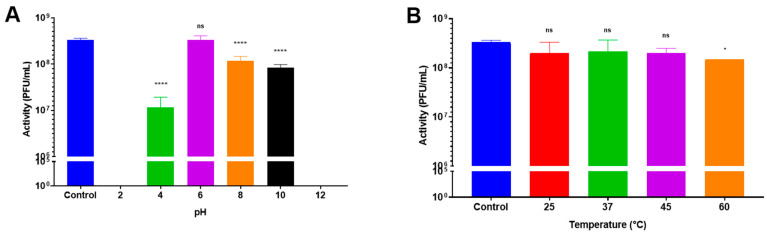
pH (**A**) and temperature (**B**) stability results of LUZ19 bacteriophages in liquid form for one hour (*n* = 3). “ns”: *p* > 0.05, *: *p* < 0.05 “****”: *p* ≤ 0.0001. For graphic A, the activity at pH 2 and pH 12 is null.

LUZ19 (our model bacteriophage) remained stable (<1 log_10_ PFU/mL reduction) for one hour, regardless of the temperature except at 60 °C (Figure 1B). In contrast, the stability of LUZ19 was strongly influenced by the pH level. Indeed, LUZ19 was not detectable at pH 2 and at pH 12 (Figure 1A). The loss in activity amounted to 1.5 log_10_ at pH 4.0 and 0.6 log_10_ at pH 10. LUZ19 remained stable at pH 6.0 with no significant loss. These results clearly showed the importance of protecting bacteriophage LUZ19 from the acidic environment of the stomach and promoting its release after the pylorus.

### 3.2. Formulation

Bacteriophage LUZ19 was spray-dried at a titer of 2.0 × 10^9^ PFU/mL (2.0 × 10^11^ PFU/mL for the stock titer) with different percentages of D-(+)-trehalose dihydrate and L-isoleucine (63.3% *w*/*w* and 36.7% *w*/*w*, respectively) and Eudragit^®^ FS-30D with PlasACRYL^®^ T20 (90.9% *w*/*w* and 9.1% *w*/*w*, respectively) weight ratios (Table 1).

**Figure 2:**
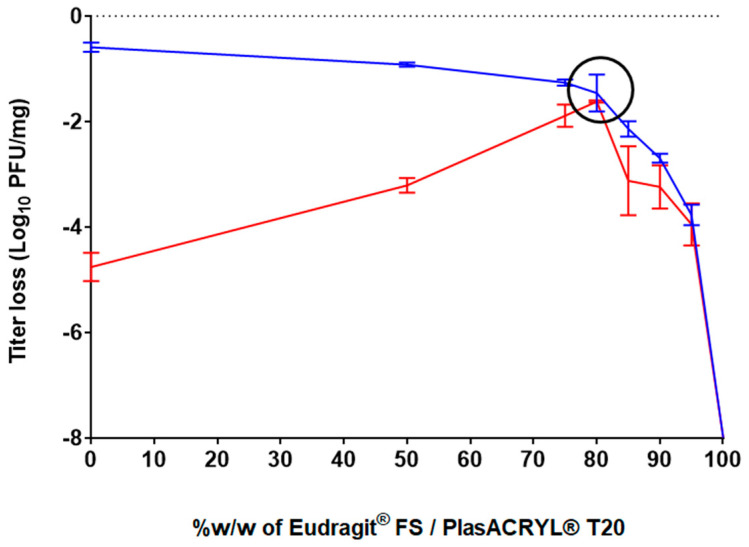
Effect of the different excipient ratios on bacteriophage survival following spray-drying and after exposure to pH = 2 for a period of 2 h (blue curve: titers after neutral resuspension with DPBS pH 7.4 showed the effect of both formulation and process; red curve: titers after acid resuspension showed the effect of the whole process of encapsulation, and the resultant acid protection).

The effect of the different ratios of these excipients on bacteriophage activity following the spray-drying process was evaluated. The spray-dried bacteriophage-containing powders were exposed to acidic conditions at pH 2.0 for 2 h and the resulting bacteriophage activity was evaluated (Figure 2). The results obtained after spray-drying for the formulation containing only D-(+)-trehalose and L-isoleucine without Eudragit^®^ and PlasACRYL^®^ T20 (formulation F1) showed a loss of activity of −0.59 log_10_ (stdev = 0.09 log_10_), which corresponded to those obtained in a previous study [40]. However, this formulation did not protect bacteriophages from acid conditions, resulting in a loss of −4.76 log_10_ PFU/mg. When the ratio of Eudragit^®^FS30D/PlasACRYL^®^ T20 was increased, the bacteriophages were less stable after spray-drying because a minimal amount of D-(+)-trehalose was needed to protect the bacteriophages from desiccation. In contrast, bacteriophage stability upon contact with the acidic medium increased due to an increase in the total percentage of Eudragit^®^ FS.

A D-(+)-trehalose/L-isoleucine to Eudragit^®^ FS30D/PlasACRYL^®^ T20 ratio of 20:80 (F4) showed a loss of activity of −1,46 log_10_ PFU/mg after processing and, interestingly, no loss of activity after 2 h at pH 2.0. Surprisingly, we observed that an increase in the percentage of Eudragit^®^ FS/PlasACRYL™ in F5, F6, and F7 was deleterious for the bacteriophage after spray-drying. The loss of activity after 2 h at pH 2.0 was due to the initial loss of activity after the spray-drying process. When using 100% *w*/*w* of Eudragit^®^ FS30D/PlasACRYL^®^ T20 (F8), LUZ19 was already totally inactivated after spray-drying.

Therefore, it can be hypothesized that even when Eudragit^®^ FS allows for the protection of LUZ-19 in an acid medium, a minimum concentration of D-(+)-trehalose was needed to protect the bacteriophages from desiccation stresses, as previously described by Vinner and co-workers [35].

In the study performed by Stanford et al., a unique formulation consisting of 10% *v*/*v* of bacteriophages, Eudragit^®^ S100, and an undescribed stabilizer with an unknown concentration was spray-dried. The encapsulation of the bacteriophages resulted in a loss of about 1 log_10_ after the process, which was comparable to the results obtained in our study, even if the atomizer used was different (rotary atomizer). However, after exposure of the powder to a pH of 3.0 for 20 min, an average recovery of only 13.6% of the bacteriophages was obtained [41]. Such data demonstrate the great efficacy of our formulation and protocol of atomization.

In the study of Vinner et al., the formulation containing only D-(+)-trehalose totally preserved the activity of the bacteriophage after the process. In contrast, our formulations containing only D-(+)-trehalose and L-isoleucine have shown a loss of activity of 0.5 log_10_. Moreover, also in contrast to our study, where a total loss of activity of LUZ19 was observed, their formulation containing only Eudragit S100^®^ showed a loss of activity of 4 log_10_ after spray-drying. Such differences are probably due to the influence of the bacteriophage itself, i.e., its resistance to the shear stresses and temperatures of the spray-drying process. Vinner and colleagues also evaluated the resistance of their bacteriophage-loaded delayed-release system at pH 2.0 and observed a loss of activity ranging from 3 to 2 logs_10_, depending on the used formulation [27].

### 3.3. Characterization of Eudragit^®^ FS-Based Microparticles

The yield of atomization decreased from 90% to 45% *w*/*w* when the percentage of Eudragit^®^ FS/PlasACRYL^®^ T20 increased (Table 1). For instance, F4, which contained 80% *w*/*w* of Eudragit^®^ FS/PlasACRYL^®^ T20, showed a yield of 70% *w*/*w*.

The moisture content of spray-dried powders ranged between 1 and 4% *w/w*. It was shown that the higher the Eudragit^®^/PlasACRYL^®^ T20 content, the drier the powder. As previously observed, a decrease in the moisture content increased the electrostatic charges that reduced the yield of the process.

The Tg (°C) was found to be inversely correlated to the RMC (%). Indeed, it is well known that the moisture content is exponentially correlated with the Tg (the higher the moisture content is, the lower the Tg is), as described in the Gordon–Taylor equation [54]. It is important to consider these parameters in view of the stability of the powder over time. Indeed, bacteriophage viability in a powder is closely associated with the temperature gap between the storage temperature (Ts) and the Tg [55]. On the other hand, when the level of Eudragit^®^ FS/PlasACRYL^®^ T20 increased, the percentage of amorphous structure in the whole spray-dried powder increased to a range between 44% (0% of Eudragit^®^ FS/PlasACRYL™ T20 in F1) and 76.8% (100% of Eudragit^®^ FS PlasACRYL™ T20 in F8). Due to the high rate of crystalline L-isoleucine, when its amount decreased from F1 to F8, the percentage of the amorphous form of the powder increased (Figures S1 and S2). The different spray-dried powders were characterized by similar particle size distributions (Table 3). The median particle sizes. were ranged between 2.5 and 3.5 µm.

**Figure 3:**
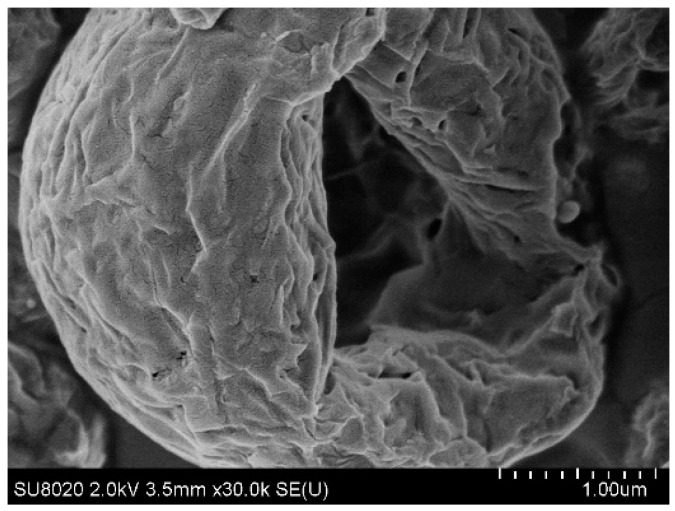
Scanning electronic micrographs of the surface of a particle containing LUZ19 encapsulated in 100:0% *w*/*w* of D-(+)-trehalose/L-isoleucine: Eudragit^®^ FS/PlasACRYL^®^ T20.

**Figure 4:**
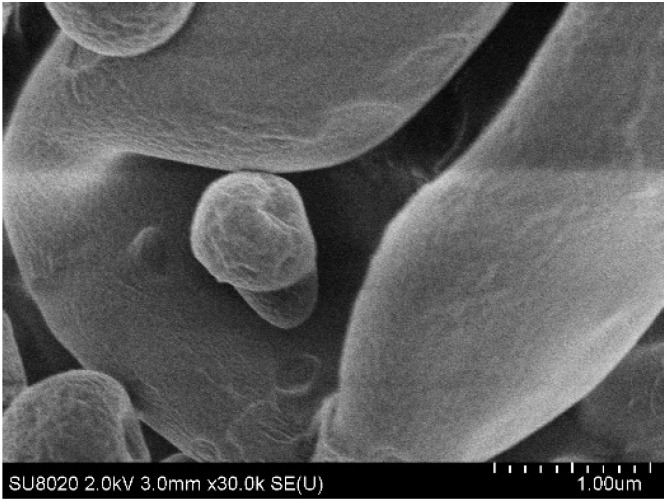
Scanning electronic micrographs of the surface of a particle containing LUZ19 encapsulated in 20:80 % *w*/*w* of D-(+)-trehalose/L-isoleucine: Eudragit^®^ FS30D/PlasACRYL^®^ T20 (F4).

Particles containing only D-(+)-trehalose and L-isoleucine (F1) were characterized by a rough surface (Figure 3). For particles containing 80% *w*/*w* of Eudragit^®^ FS30D/PlasACRYL^®^ T20 (F4), the surface was clearly smoother (Figure 4). Being in the form of a latex dispersion composed of soft nanoparticles, which may fuse during the atomization, an increase in the percentage of Eudragit^®^ FS produced a smoother aspect of the bacteriophage-loaded microparticles.

### 3.4. Characterization of the Flowability

The flowability of formulations F1 and F4 were characterized to evaluate the influence of Eudragit^®^ FS on flowability, as F1 did not contain the colonic-targeting polymer. Both powders were characterized as “very, very poor”, according to the European Pharmacopeia criteria [Ph.Eur.10.0,20936(01/2010)] (Table 4) with a better flowability for formulation F4, containing Eudragit^®^ FS (e.g., lower Carr’s index and Hausner ratio). The size and density of the particles being similar, this difference could be due to the smoother surface of these particles (F4) (Figure 4).

### 3.5. Capsules Filling

Capsules of 3 different sizes (00, 0 and 1) were filled with F1 and F4 spray-dried powders. A mass uniformity test of the capsules was carried out to ensure the homogeneous filling of the capsules (Table 5).

The powder only composed of D-(+)-trehalose -L-isoleucine (F1) was more hygroscopic and its flow in the capsules was less fluid than the powder containing Eudragit^®^ FS and PlasACRYL^®^ (F4). Therefore, colonic-release encapsulated microparticles offer a seductive alternative to the use of gastro-resistant capsules filled with unprotected spray-dried powders (F1). Indeed, powder (F4) containing Eudragit^®^ FS has already shown its ability to protect the bacteriophage from acidity. Standard capsules, which are commonly used in compounding pharmacies, are suitable. The use of standard capsules, which are commonly used in magisterial preparations (compounding pharmacies), facilitates the implementation of personalized or precision bacteriophage therapy approaches, which are promoted by several researchers [56]. Such capsules can also be opened to disperse the colon-targeted bacteriophage-loaded powder in water for patients with swallowing issues.

### 3.6. Characterization of the Mix for Tableting

In addition to their use in capsules, the developed spray-dried powders may also be incorporated into tablets. This dosage form cannot be produced in hospitals or compounded in pharmacies, but minitablets may be used for personalized medicine. Moreover, it was shown that our spray-dried bacteriophage-loaded powders exhibited poor flowability. Therefore, carriers had to be added to assure the proper flowability of the mixture (Table 2 and Table 6). For instance, the flowability of the mixture containing F4 increased from very poor to passable, according to the European Pharmacopeia [Ph.Eur.10.0,20936(01/2010)] criteria.

The physical properties of the spray-dried microparticles produced using formulation F4 and the excipients dedicated for compression (Table 2) allow for the production of robust tablets.

### 3.7. Characterization of Tablets

The average mass of the tablets was found to be 155 ± 2 mg. Conforming with the European Pharmacopoeia criteria for tablet mass uniformity, the individual mass of not more than 2 of the 20 tablets deviated from the average mass to a higher percentage than 10%, and the mass of any unit did not deviate by more than double that percentage. In addition, the loss of mass after the friability test was 0.9% *w*/*w*, which was below the recommended threshold loss of 1%. The tablets’ hardness was found to be 112 ± 8.4 N, and their disintegration time was evaluated at 2 min 34 sec in DPBS and 5 min 6 sec in HCl 0.1N, which was lower than the conventional 15 min that is allowed for standard tablets.

The F4 powder was evaluated in terms of acid stability after tableting. No significant difference before and after tableting was observed.

### 3.8. Activity Release Test of Bacteriophage LUZ19 from Powder Using a Simulator of the Human Intestinal Microbial Ecosystem (SHIME^®^) Model

**Figure 5:**
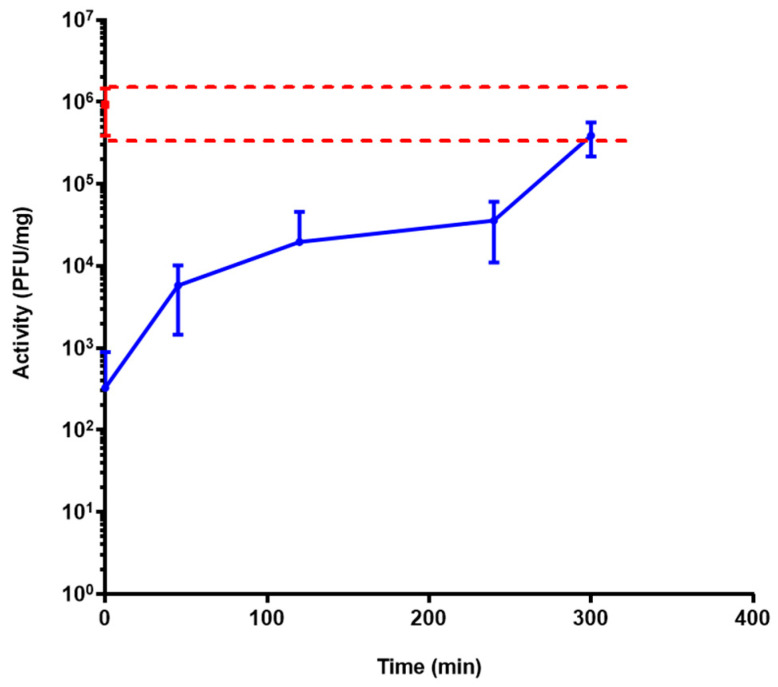
Liberation profile of LUZ19 encapsulated (F4) at pH > 7 for 5 h (blue curve). The red dashed lines represent the low and high limits of activity of the control (powder whose activity was tested with resuspension in DPBS).

The release profile of formulation F4 was assessed, in triplicate, with three bioreactors. After the incubation of the formulation in the feed for 2 h, 60 mL of PJ was automatically added in each reactor for 15 min (which gradually conducted the solution to pH 7 and was forced at pH 7.4). At pH 5 and 6, no bacteriophage activity was observed. At pH 7.4, the release of active LUZ19 bacteriophages started and was sustained over 5 h. At pH levels lower than 7.0, the enteric polymer was insoluble and prevented the release of the bacteriophage. At pH 7.4, the polymer started to dissolve, which initiated the release of LUZ19. The release was sustained for more than 5 h, as a result of the progressive erosion of the dosage form (Figure 5). Our data show that our spray-dried system was a solid dispersion in which the bacteriophage was entrapped in a matrix consisting of the enteric polymer.

The profile of dissolution observed in the SHIME^®^ model, in terms of acid resistance and transit time, should in theory allow for the decolonization of bacterial pathogens all along the ileum, cecum, and proximal colon.

### 3.9. Storage Stability Tests

**Figure 6:**
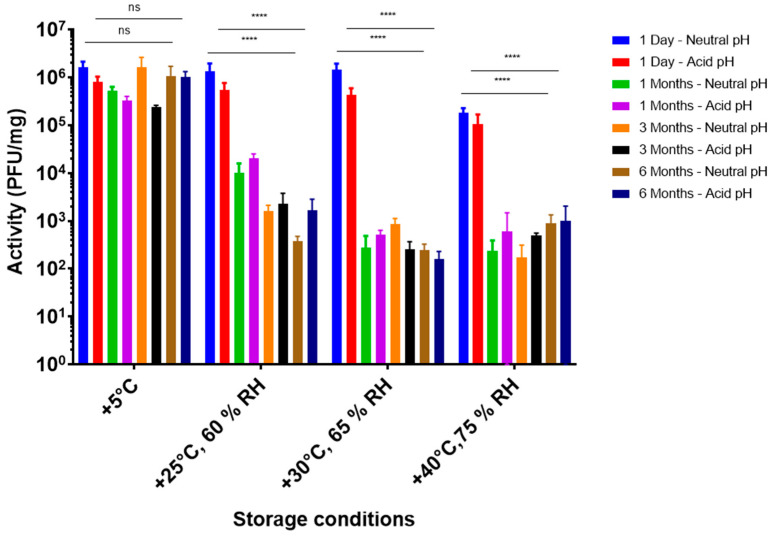
Activity (PFU/mg) of the bacteriophages in F4 powder according to storage temperature, relative humidity (HR), and time “ns”: *p* > 0.05 and “****”: *p* ≤ 0.0001.

A storage stability study at different ICH conditions was conducted with F4, the most promising formulation in terms of acid stability (Figure 6).

At +5 °C, no significant difference in bacteriophage activity was observed over a 6-month period and this accounted for the direct dissolution of powder in DPBS at pH 7.4 (neutral), as well as after 2 h at pH 2 (acid). Therefore, it was concluded that encapsulated LUZ19 powders, using formulation F4, were stable at +5 °C over 6 months. Moreover, no deterioration of the acid protection capacity over time was observed when stored at 5 °C. However, at higher temperatures, the activity decreased considerably after 1 month of storage, without a further decrease for up to 6 months. As there was no significant difference between neutral and acidic conditions, it was hypothesized that there was no deterioration of the enteric polymer. The loss of bacteriophage activity was most likely due to the lack of D-(+)-trehalose and L-isoleucine compared to the formulation without Eudragit^®^ FS and PlasACRYL [40]. The stabilization at 6 months and +5 °C was encouraging.

From an industrial point of view, spray drying is widely described to be a fast, reliable, and cost-effective method. Moreover, the developed formulation has shown long-term stability at 5 °C. From a clinical point of view, these powders are very interesting as they could allow the pharmacist within the hospital to make compounding preparations with different bacteriophages. This would allow a rapid personalized oral treatment that is still considered to be the route of administration offering the highest compliance for patients.

## 4. Conclusions

Eudragit^®^ FS microparticles containing bacteriophages were successfully produced through spray-drying. The developed formulation yielded a bacteriophage-loaded powder that effectively protected the bacteriophages from gastrointestinal acidity, exhibiting a reduction of only −1.46 log10 PFU/mg after the spray-drying process. The particles demonstrated a mean diameter (Dv50) of 2.97 µm. In the SHIME^®^ model, it was demonstrated that the delivery of bacteriophages was delayed and prolonged for up to 5 h at pH > 7.4, theoretically enabling the targeted disinfection or decolonization of bacterial pathogens throughout the ileum, cecum, and proximal colon of patients. Importantly, the powder exhibited stability for a minimum of six months when stored at +5 °C and could be administered orally in various forms, such as extemporaneously dispersed powder, capsules, or tablets. To further validate these promising in vitro results, an in vivo study should be conducted.

## Figures and Tables

**Table 1:** Formulations of the microspheres. Initial mixture D-(+)-trehalose/L-isoleucine—63.3/36.7% *w/w*; initial mixture Eudragit^®^ FS/PlasACRYL^®^—90.9/9.1% *w*/*w*.

Formulations	% *w*/*w* D-(+)-Trehalose and L-Isoleucine	% *w*/*w* Eudragit^®^ FS-30D with PlasACRYL^®^ T20
F1	100	0
F2	50	50
F3	25	75
F4	20	80
F5	15	85
F6	10	90
F7	5	95
F8	0	100

**Table 2:** Details of the tableting mix.

Excipients/Formulations	% *w*/*w*
Spray-dried powder	3.00
Microcrystalline cellulose	55.48
Mannitol	36.39
Colloidal silicon dioxide	0.10
Hydroxypropyl cellulose	2.42
Croscarmellose sodium	1.61
Magnesium stearate	1.00
TOTAL	100.00

**Table 3:** Particle size analysis of different powders containing LUZ19 encapsulated.

Formulation		Dv10 (µm)	Dv50 (µm)	Dv90 (µm)
F1	Average	0.87	2.54	6.27
	Standard deviation	0.00	0.04	0.22
F4	Average	1.19	2.97	6.64
	Standard deviation	0.00	0.02	0.09

**Table 4:** Characteristics regarding flowability of the non-encapsulated (F1) and the optimally encapsulated (F4) powder containing LUZ19 (ρP: Particle density; ρL: Loose bulk density; ρT: Tapped bulk density; ε: Porosity; CI: Carr’s index; HR: Hausner ratio).

Formulation		ρ*P* (g/cm^3^)	ρ*L* (g/cm^3^)	ρ*T* (g/cm^3^)	*ε* (%)	CI (%)	HR
F1	Average	1.3891	0.1093	0.3080	77.82	64.51	2.82
	Standard deviation	0.0062	0.0029	0.0047	0.13	0.41	0.03
F4	Average	1.3664	0.1790	0.3016	77.93	40.65	1.68
	Standard deviation	0.0062	0.0070	0.0139	0.12	0.47	0.01

**Table 5:** Mass uniformity of capsules filled with F1 and F4.

	F1			F4		
Capsules size	00	0	1	00	0	1
Average mass of powder (g)/capsule	0.269	0.164	0.156	0.312	0.265	0.250
Standard deviation	0.008	0.008	0.008	0.008	0.009	0.007
Lower limit (g)	0.249	0.148	0.140	0.289	0.239	0.225
Upper limit (g)	0.289	0.181	0.171	0.335	0.392	0.275
Compliance	Compliant	Compliant	Compliant	Compliant	Compliant	Compliant

**Table 6:** Characteristics regarding flowability of the mix containing encapsulated powder containing LUZ19 (ρP: Particle density; ρL: Loose bulk density; ρT: Tapped bulk density; ε: Porosity; CI: Carr’s index; HR: Hausner ratio).

Formulation		ρ*P* (g/cm^3^)	ρ*L* (g/cm^3^)	ρ*T* (g/cm^3^)	*ε* (%)	CI (%)	HR
F4+mix	Average	1.5407	0.4336	0.5781	62.48	25.00	1.33
	Standard deviation	0.0054	0.0026	0.0033	0.12	0.82	0.01

## Supplementary Materials

The following supporting information can be downloaded at: https://www.mdpi.com/article/10.3390/pharmaceutics15061602/s1, Figure S1. XRD profiles of spray-dried (A): trehalose, (B): L-Isoleucine, (C) Eudragit FS30D and (D) Plasacryl T20; Figure S2. XRD profiles of formulations: F1 (blue), F2 (red), F3 (green), F4 (purple) and F5 (orange).
