# Supplementary material for: Eudragit® FS Microparticles Containing Bacteriophages, Prepared by Spray-Drying for Oral Administration
Source: Pharmaceutics. 2023 May 27;15(6):1602. doi: 10.3390/pharmaceutics15061602 (PMC10305712; doi:10.3390/pharmaceutics15061602)
Supplement: Supplementary file 1 [file pharmaceutics-15-01602-s001.zip › pharmaceutics-2404349-supplementary.pdf]

Supplementary Material

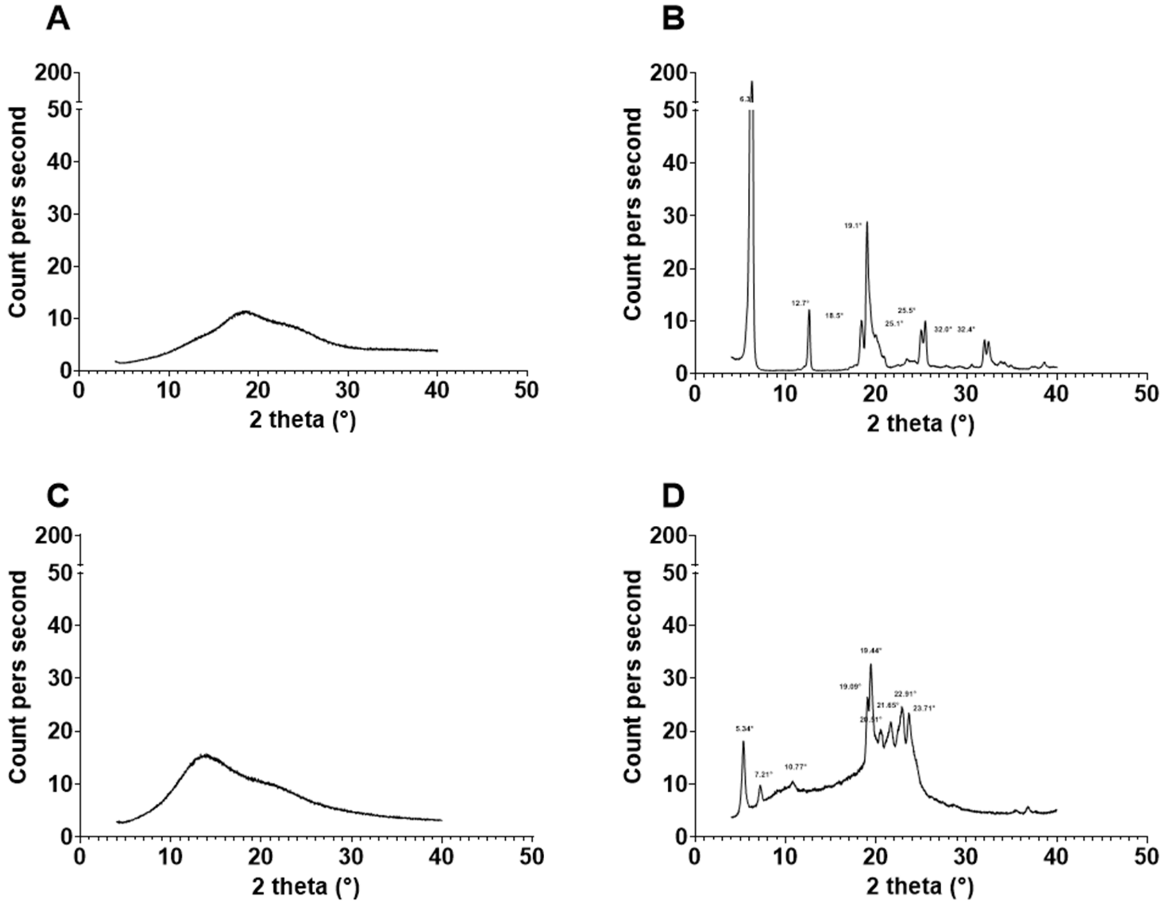

Figure S1.XRD profiles of spray-dried (A): trehalose, (B): L-Isoleucine, (C) Eudragit FS30D and (D) Plasacryl T20.

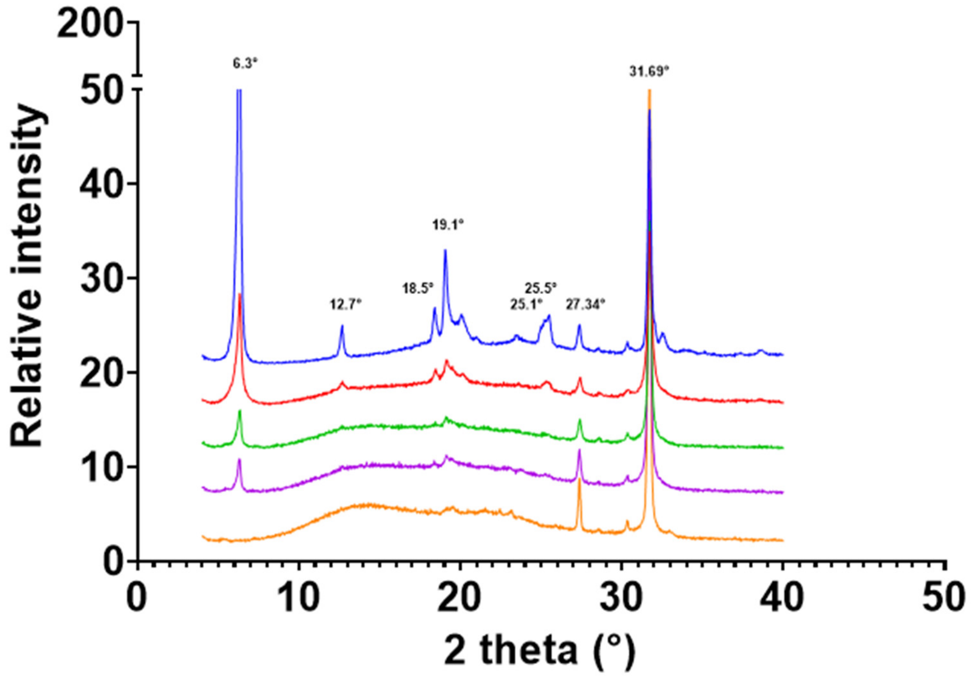

Figure S2. XRD profiles of formulations: F1 (blue), F2 (red), F3 (green), F4 (purple) and F5 (orange).
